# The Prevalence of Multiple Sclerosis in 3 US Communities: The Role of Vitamin D

**Published:** 2010-06-15

**Authors:** William B. Grant

**Affiliations:** Sunlight, Nutrition, and Health Research Center, San Francisco, California

## To the Editor:

Authors of the recent article on the prevalence of multiple sclerosis (MS) in 3 US communities (1) presented excellent findings but stumbled on their interpretation. The article noted that the findings were similar to those in a study of MS prevalence from the 1940s and 1950s (2); however, the authors did not make a detailed comparison with those data. Furthermore, the authors noted that UV radiation could explain the findings but did not suggest a mechanism for the link.

The data in reference 2 have been analyzed in previous studies, which found the distribution of MS in the United States to be well described by a quadratic fit to latitude (3). This fit is related to wintertime solar UVB (4). Winter is the season with the lowest levels of serum 25-hydroxyvitamin D in humans and the highest rate of viral infections (4). In a comparison of the prevalence data from reference 1 with those from reference 2, the recent data indicate a prevalence of approximately 7 cases per 100,000 per year less but with the same functional fit (Figure). The quadratic fit to the data in reference 2 explains 75% of the variance.

**Figure.**
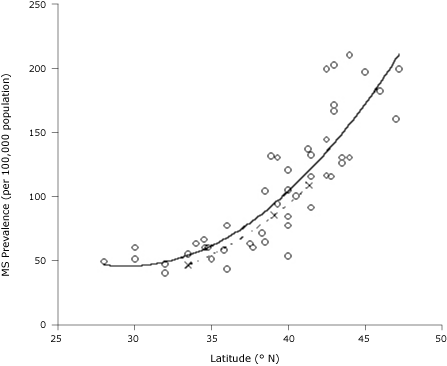
Prevalence of multiple sclerosis (MS) by latitude in the United States according to data from reference 1 (×) and reference 2 (o). The dashed line is a quadratic fit to the data in reference 1, and the solid line is a fit to the data in reference 2.

The most likely role of UVB in reducing risk of MS is through production of vitamin D. The Epstein-Barr virus has strong support as a possible risk factor for MS, as does vitamin D in reducing risk (5). Progress is being made in understanding the mechanisms by which vitamin D reduces the risk of MS (6). Among its other beneficial properties, vitamin D strengthens the innate immune system by inducing production of cathelicidin and defensins, both of which have antimicrobial and antiendotoxin effects (4-6).

Given that the role of vitamin D in reducing the risk of MS was first described in 1974 (4), it is disappointing that recommendations to increase serum 25-hydroxyvitamin D levels, especially in winter, have not been made more forcefully by US, European, and Australian health officials. The Institute of Medicine of the National Academies is scheduled to announce new dietary reference intakes for vitamin D and calcium in mid-2010 (www.iom.edu/Activities/Nutrition/DRIVitDCalcium.aspx), so I hope this shortcoming will be rectified soon.

Read the author's reply
